# A Report of the Royal Jennerian Society, on the Supposed Failures of Vaccination at Ringwood, in Hampshire

**Published:** 1808-04

**Authors:** 


### *A Report of the Royal Jennerian Society, on the supposed Failures of Vaccination at Ringwood, in Hampshire.*

Salisbury Square, Feb. 3, 1808.

THE Royal Jennerian Society, deeply impressed with the importance of their pledge to the public, in recommending Vaccination as a security against the Small-pox, and feeling equally the claim the public have on them to justify this pledge by offering such information as may remove any reasonable doubt respecting this security, think it their duty to publish an abstract of their proceedings, in consequence of the alarm excited by the supposed failures of vaccination at Ringwood.

Upon information received from the Right Honourable George Rose, M. P. to whom the Society are greatly indebted for his zeal and attention on this interesting occasion, the Society appointed a medical deputation, consisting of John Ring, Esq. Vice President, W. Blair, Esq. Director, and Dr. J. S. Knowles, their Resident Inoculator.

These gentlemen, assisted by Dr. Fowler, an eminent physician of Salisbury, who is totally unconnected with this Society, proceeded to Ringwood; where a public meeting was convened at the Town Hall, and attended by the Right Honourable George Rose, W. Mills, Esq. M. P. S. Tuncks, Esq. a Magistrate of the town, the Rev. Dr. Taylor, the Rev. Mr. Davies, the Rev. Mr. Middleton, Mr. Westcott and Mr. Macilwain, Surgeons of Ringwood, and the other principal inhabitants of that town and neighbourhood. In their presence the medical gentlemen, during two whole days, went into a close investigation of these supposed failures of Vaccination.

Their report (which is open to the inspection of any medical man) affords the most consolatory results. These general results the Society now lay before the public, to defeat the effects of prejudice or misrepresentation, and to confirm the efficacy and advantage of Dr. Jenner's great discovery, *the Cow Pock Inoculation,* as a safe, mild, and *uncontagious* antidote against that most terrible and *contagious* malady the small-pox.

On the whole, the Medical Deputation are perfectly satisfied, after a minute and careful examination of the numerous cases brought before them, that no instance occurred, during the dreadful visitation at Ringwood, of the small-pox having taken place where the process of Vaccination had been complete; and they have the highest satisfaction in offering to the public a confirmation of their own opinion, in the subjoined communications from the two medical practitioners at Ringwood, by whom the majority of the inhabitants were inoculated.

### *General Result of the Inquiry into the unfavourable Reports concerning Vaccination at Ringwood.*

The small-pox appeared at Ringwood about the middle of September; and rapidly spread through the town and neighbourhood, partly by means of inoculation, and partly by natural infection.

Vaccine inoculation did not commence until the 23d of October; it is therefore evident, that all those persons who were vaccinated, had been previously exposed to the contagion of the small-pox.

Some of these persons had the small-pox at the same time with the cow-pock, in consequence of previous infection. In others, vaccine inoculation did not take effect; and consequently they were not rendered insusceptible of the infection of the small-pox.

In various instances, dry cow-pock matter, received from several quarters, was dissolved in water almost boiling, previous to insertion; and it is probable, that on this account it frequently failed to produce any effect. Above two two hundred persons, however, were successfully vaccinated; and have been protected from the small-pox, though much exposed to its infection in different ways.

It was asserted, that the small-pox was more fatal, at Ringwood and the neighbouring villages, to those persons who were inoculated for the cow-pock, than to others. This report appeared to be totally destitute of foundation. The mortality was indeed considerable, owing in some instances to want of air and cleanliness, and in others to the immoderate use of spirituous liquors, particularly at the time of the eruption, which had been recommended by a Thresher, who inoculates for the small-pox.

It was reported, that several persons at Ringwood, who were inoculated with the cow-pock some years ago, lately had the small-pox: but no satisfactory evidence was given to establish the fact; as it appeared either that their arms had not been inspected by the inoculator after vaccination, or that there was no proper scar left behind; or, on the other hand, when they were put to the test of variolous inoculation, no other effect was produced, than what is occasionally produced in those who have previously had the small-pox.

It was also insidiously reported that two persons died of the cow-pock (or it has been termed, the "Vaccine ulcer"): but it is positively asserted by the surgeons who inoculated them, that no vaccine ulcer, nor cow-pock, took place in either of those instances; and that the patients died of other diseases—one of them of an apoplexy.

JOHN RING.
WILLIAM BLAIR.
J. S. KNOWLES.

The preceding Report having been submitted to Dr. Fowler, an answer dated *(dated Sarum, Jan.* 31,) has been received, in which he says, "I perfectly approve of this Report; as it very accurately expresses the opinion which I have formed, of the causes of the supposed failures of Vaccination at Ringwood." Mr. Rose has likewise permitted the Jennerian Society to add, "that he has seen this Report, and concurs in it, so far as he as is able to form a judgment on the subject."

Printed by Order of the General Court,
CHARLES MURRAY,
Secretary.

*Extract*

*Extract of a Letter from Mr. Westcott to Mr. Blair, dated Ringwood, Jan.* 10, 1808.

"Mr. Birch must now be convinced by my answer to his letter, that his statement is *directly wrong*, respecting the failures of Vaccination at Ringwood; and you are at perfect liberty to make use of my name, in any manner you may think proper, to convince the world that Mr. Birch has asserted a falsehood."

*Copy of a Letter from Mr. Westcott to Mr. Ring, dated Ringwood, Jan.* 15, 1808.

Dear Sir,

I am of opinion that not one person in Ringwood or its neighbourhood, caught or had the Small-pox after going through regular and complete Vaccination.

I remain, dear Sir, your obedient Servant,

W. WESTCOTT.

P. S. Yours would have been answered sooner, but I could not see Mr. Macilwain till last evening. He says, these are exactly his sentiments.

*Copy of a Letter from Mr. Macilwain to Mr. Ring, dated Ringwood, Jan.* 25, 1808.

Dear Sir,

In anwer to your letter, which was dated 21st instant, but which I only received on Saturday the 23d, I have to inform you that the resolution which appeared in the Salisbury and London papers, respecting the vaccination here, contained my sentiments, and that I have no reason to alter my opinion at present. The advertisement I allude to is the following:

"After a most careful and minute investigation of those cases in which the small-pox occurred subsequently to inoculation for the cow pox, it appeered, that such inoculation had not taken effect, or that when an effect had been produced, the progress of vaccination was interrupted, so as to render the patients insecure.

"The result cannot fail to be highly interesting to the inhabitants of Ringwood, and of the neighbouring parishes; inasmuch as it must remove the feeling of alarm which had been excited, and restore and confirm the confidence of the public in a practice affording protection against a pestilential disease, justly esteemed the scourge of the human race.

"The investigation was made in the presence of some of the most respectable gentleman of the town and neighbourhood, by Dr. Fowler of Salisbury, and a deputation tion of three members of the Royal Jennerian Society of London."

I proposed to *re-vaccinate* many persons with the matter you were so kind to give me, but I only used it in two instances, IN BOTH OF WHICH IT SUCCEEDED.

I cannot say more to you on the subject of Vaccination, than I did when you were at Ringwood. I consider it as an inestimable blessing; and solemnly and seriously am of opinion, that it is a preventive and effectual preservative against the Small-pox, when carefully conducted: and if the people of Ringwood had allowed themselves to have been fairly and honestly informed of its merits, the lives of many would have been saved, and the malicious intentions of some persons in this quarter, to stigmatize the Jennerian system, would have been defeated.

The enemies of Vaccination did all they could to propagate the Small-pox among those who were desirous of the Cow-pox; and the people were much too incautious to give the new inoculation any thing like a fair chance.

If any thing worth communication should occur, I shall very gladly avail myself of your desire to hear from me. In the mean time,

I am, dear Sir, your most obedient Servant,

G. MACILWAIN.

